# The community acquired pneumonia endotypes and phenotypes dataset

**DOI:** 10.1038/s41597-025-05963-0

**Published:** 2025-10-24

**Authors:** Natalia Sanabria-Herrera, Esteban Garcia-Gallo, Sara Duque-Vallejo, Cristian Serrano-Mayorga, Ingrid Gisell Bustos, Julián Lozada, Lina Méndez-Castillo, Erica Y. Garcia-Garcia, Luis Felipe Reyes

**Affiliations:** 1 Clínica Universidad de la Sabana, Chía, Colombia; 2 ISARIC, Pandemic Sciences Institute, University of Oxford, Oxford, UK; 3 Unisabana Center for Translational Science, School of Medicine, Universidad de La Sabana, Chia, Colombia; 4 Biosciences PhD., Engineering faculty, Universidad de La Sabana, Chía, Colombia

**Keywords:** Translational research, Databases, Infectious diseases

## Abstract

The *Community-Acquired Pneumonia, Endotypes, and Phenotypes* (NACef) Dataset is a single-center dataset that includes clinical information from 768 patients diagnosed with Community-Acquired Pneumonia (CAP) at Clinica Universidad de La Sabana, Colombia. CAP continues to be a prevalent infectious condition linked to high morbidity and mortality rates during hospitalization. To help construct knowledge around this condition, this dataset encompasses Baseline Clinical Data, In-hospital follow-up, and post-hospital discharge information. This repository was captured in a prospective research study, constituting an observational cohort in translational science medicine, as we also collected biological samples to dissect the underlying mechanisms associated with severe CAP. This dataset provides opportunities for diverse statistical analyses and educational initiatives, since it contains robust clinical data and laboratory results from patients diagnosed with CAP, including data regarding COVID-19 infection. Being the first Colombian clinical database available on PhysioNet, it will contribute to a better understanding of CAP, its endotypes, and phenotypes in low- and middle-income countries (LMIC).

## Background & Summary

Community-acquired pneumonia (CAP) is the most prevalent lower respiratory tract infection worldwide^1^. Over one million patients per year require in-hospital treatment due to CAP^2^. Furthermore, it is the leading cause of hospitalization and in-hospital mortality globally^3^. It is the fourth-leading cause of death worldwide and the second-leading cause of death in low- and middle-income countries^4^. The yearly economic burden of CAP exceeds 10 billion euros in Europe and 17 billion dollars in the USA^5–7^. Despite global interventions, CAP remains a growing healthcare problem, with mortality rates ranging from 20% to 50%^8^. Therefore, continued research into CAP is essential to explore new measures to benefit the population.

Identifying the microorganism causing CAP is achieved in only 30% of cases, and the choice of empiric antimicrobial treatment remains difficult despite the widespread adoption of international guidelines^3,8–10^. However, the development of microbiological isolation techniques has improved the characterization of microorganisms commonly associated with pneumonia^11,12^, facilitating clinicians’ decision-making. Despite improvements in understanding pneumonia and the development of high-quality treatments, patients hospitalized with CAP continue to face a high prevalence of antibiotic-resistant microorganisms and elevated mortality rates^9,10,13^.

Given the epidemiological shift observed in the incidence rates of CAP following the COVID-19 pandemic^14,15^, the inclusion of patients with suspected or confirmed COVID-19 was considered pertinent when constructing this database. This approach aims to evaluate the comparisons of these respiratory infectious diseases within this population. This database behaves as a unique resource for improving the understanding of disease-specific mechanisms and as a valuable clinical and epidemiological tool for research.

Therefore, it is imperative to continue studying the clinical profiles—endotypes, phenotypes, and biomarkers—resulting from infections by specific microorganisms to adapt treatments more precisely for each patient. This dataset offers a comprehensive resource for improving our understanding of CAP, including cases with COVID-19 diagnosis. By integrating clinical, demographic, and biomarker data to identify distinct endotypes and phenotypes, this dataset becomes a valuable resource for improving translational medicine and holds significant potential to perform medical and scientific research, to guide applications in clinical practice. This contribution to the international research community underscores the importance of sharing high-quality clinical data to enhance our understanding of infectious diseases and improve patient care. The purpose of this data descriptor is to detail the data collection, processing, and structure of the database, ensuring its usability and reproducibility for the scientific community.

## Methods

### Data collection and curation

Recruitment was conducted without external advertising, relying on real-time screening of eligible patients by trained research staff. The inclusion criteria were patients over eighteen years old, with a clinical picture compatible with CAP according to the American Thoracic Society and Infectious Diseases Society of America (ATS/IDSA) definition^16^ and who were diagnosed within the first 24 hours of their hospital stay. Exclusion criteria were inability to consent, patients with active tuberculosis, diagnostic doubt, or greater likelihood of a differential diagnosis. Clinical data of patients admitted to a third-level hospital in Chía, Colombia, diagnosed with CAP, was collected using REDCap^17,18^ (Research Electronic Data Capture).

Patients were recruited from the emergency department between January 2020 and July 2022. A total of 768 patients were included in the study. Although the number of patients initially screened for eligibility was not systematically recorded, all including patients fulfilled the diagnostic criteria for CAP and were identified within the first 24 hours after admission. Trained research staff conducted daily reviews of all admitted patients to identify those meeting eligibility criteria, ensuring a systematic and prospective enrolment process. The database comprises 83 variables derived from three structured questionnaires completed for each participant, focusing on clinical and paraclinical data, the patient’s general context, prior history of antibiotic use, and severity scores such as SOFA (Sequential Organ Failure Assessment score), CURB-65, and PSI (Pneumonia Severity Index score). Tables 1–3 provide a detailed breakdown of these variables.

**Table 1:** Baseline variables, summary statistics, and missing data in the dataset.

Identification and Baseline			
Characteristic	Variable name	Summary statistics	Missing data
Patient age in years (median, IQR)	age	60 (49–70)	0 (0)
Gender, male (%)	gender male	482 (62.8)	0 (0)
Height (cm) (median, IQR)	height	165 (158–170)	361 (47.0)
Weight (Kg) (median, IQR)	weight	74 (64.2–84)	278 (36.2)
Body Mass Index (median, IQR)	BMI	27.2 (24.4–30.2)	364 (47.4)
Patient is a health worker (%)	health_work	4 (0.5)	0 (0)
Patient work in livestock or meat industry (%)	rural_work	3 (0.4)	0 (0)
Patient lives in a geriatric home (%)	geriatric_home	11 (1.4)	0 (0)
Patient in a state of prostration (%)	postramiento	10 (1.3)	0 (0)
Patient lives in overcrowding (%)	living_space	4 (0.5)	0 (0)
Previous influenza vaccination (%)	vac_influenza	10 (1.3)	63 (8.2)
Previous pneumococcus vaccination (%)	vac_neumococo	2 (0.3)	63 (8.2)
Respiratory infections in the last 12 months (%)	prev_infec	27 (3.5)	1 (0.13)
Number of respiratory infections in the last 12 months (median, IQR)	num_prev_infec	1 (1-1)	—
Visits to the emergency department in the last 12 months (%)	urg_12_month	88 (1.1)	—
Number of emergency department visits in the last 12 months (median, IQR)	num_urg	1 (1-1)	—
Patient has received antibiotics in the last 12 months (%)	ab_12month	79 (10.3)	4 (0.5)
Number of antibiotics received in the last 12 months (median, IQR)	prev_ab_num	1 (1-1)	—
Number of days using antibiotics (median, IQR)	prev_ab_days	5 (3–7)	—
COPD history (%)	copd	82 (10.7)	—
GOLD classification (median, IQR)	gold	2 (2–4)	—
History of smoking (%)	ant_taba	141 (18.4)	1 (0.13)
Patient admitted to the ICU (%)	icu	261 (33.9)	234 (30.5)
COVID diagnosis (%)	sosp_covid	623 (81.1)	2 (0.3)
COVID vaccine (%)	vac_covid	88 (11.5)	7 (0.9)
Patient has severity criteria according to IDSA/ATS (%)	criteria	363 (47.3)	1 (0.13)
Meets with severity criteria according to IDSA/ATS (1 major or 3 minor) (%)	sev_criteria	254 (33.1)	3 (0.4)
SOFA scale at admission (median, IQR)	admission_sofa	2 (2–4)	30 (3.9)
CURB 65 at admission (median, IQR)	admission_curb	1 (0–2)	3 (0.4)
PSI at admission (median, IQR)	admission_psi	76 (57–100)	13 (1.7)

**Table 2:** In hospital variables, summary statistics, and missing data in the dataset.

In-hospital follow-up			
Characteristic	Variable name	Summary statistics	Missing dat a
Hospital setting (median, IQR)	hosp_stay	1 (1–2)	22 (2.9)
Patient received empiric antibiotic (%)	ab_empiric	451 (58.7)	9 (1.2)
Patients received more than one empiric antibiotic (%)	ab_conjug	263 (34.2)	66 (8.6)
Patient presented coinfection by another microorganism (%)	coinfection	74 (9.6)	68 (8.8)
SOFA scale after 72 hours (median, IQR)	sofa_72	2 (1–5)	105 (13.7)

**Table 3:** Discharge variables, summary statistics, and missing data in the dataset.

Discharge			
Characteristic	Variable name	Summary statistics	Missing data
Etiological Pathogen was identified (%)	etio_pneumo	603 (79)	2 (0.3)
Second RT-PCR test if suspected COVID-19 (%)	2_rt_pcr_covid	51 (6.6)	9 (1.2)
Patient was extubated (%)	extub	95 (12.3)	120 (15.6)
Patient required tracheostomy (%)	traqueost	24 (3.1)	672 (87.5)
Patient was discharged alive (%)	live_discharge	579 (75.3)	3 (0.4)
Patient died at the ICU (%)	icu_death	163 (21.2)	4 (0.5)
Patient died at general hospitalization (%)	gen_hosp_death	31 (4.0)	12 (1.6)
Number of days with antibiotic (median, IQR)	days_ab	6 (3–8.5)	—
Patient required mechanical ventilation during ICU (%)	ventilation	318 (41.4)	59 (7.7)
Patient had a non-pulmonary infection at the same hospital visit (%)	non_pulm_infec	94 (12.2)	9 (1.2)
Patient had any cardiovascular complication (%)	cv_comp	85 (11.1)	14 (1.8)
Patient was participating in another study (%)	another_study	129 (16.8)	0 (0)

**Fig. 1:**
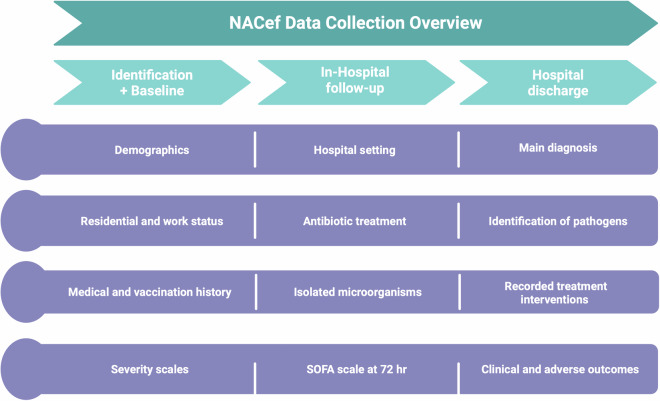
Data collection overview. This figure provides a comprehensive overview of the study’s data collection process. It details the methods used to gather patient information, including the types of data collected and the timeline for data collection. The figure highlights the structured approach employed to ensure the accuracy and reliability of the data, which forms the foundation for subsequent analyses.

The database comprises variables from various time points (Fig. 1). Initially, data were collected at Baseline (Table 1), encompassing demographics (e.g., age, gender), residential and work status (e.g., health worker, rural worker, geriatric home resident), vaccination history (e.g., previous influenza vaccination, previous pneumococcal vaccination), medical history (e.g., prior respiratory tract infections, previous emergency department visits, prior hospitalization, comorbidities, smoking history), and severity scales at admission (SOFA, CURB-65, PSI). Subsequently, data were gathered during In-hospital follow-up (Table 2), including variables such as the hospital setting where the patient was admitted, antibiotic treatment administered during hospitalization, identification of isolated microorganisms, and a follow-up SOFA scale at 72 hours post-admission. Finally, data were collected at patient discharge (Table 3), including the definitive primary diagnosis, identified pathogens, interventions during hospitalization, and various clinical and adverse outcomes.

All data were subjected to a standardized curation process to check for accuracy and consistency. This involved automated validation scripts to check for missing values and inconsistencies, followed by manual revision by clinical experts. For patient privacy, the dataset was de-identified to HIPAA standards, removing all direct identifiers. To safeguard the confidentiality of participants within the dataset, particularly concerning variables such as age, weight, and height, histograms were generated to identify and categorize outliers. This process was fundamental, as identifiable values can potentially lead to the disclosure of individual identities. To mitigate the risk of identifying patients based on registration dates, datetime objects were generated, and the difference in days for each date relative to the admission date was calculated for each date variable in the dataset. This randomized adjustment ensures the confidentiality of precise registration dates, thereby improving the overall privacy of the dataset.

The diagnosis of CAP by the treating physicians based on clinical, radiologic, and microbiologic criteria, according to international guidelines. While there was no independent adjudication committee established, several steps were taken not to allow diagnostic heterogeneity: (1) application of similar criteria in identifying CAP; (2) evaluation of patients in a multidisciplinary environment by infectious disease physicians, internal medicine specialists, and intensivists in the course of regular clinical assessment; and (3) advanced microbiologic and immunologic typing by using real-time PCR, genomic, proteomic, and metabolomic studies, with the capability to improve case correctness in classification as well as in differentiating between subgroups.

The historical and clinical variables in this dataset, including comorbidities and risk factors, were screened manually from clinical records; such factors were encoded as binary (0/1) whenever well-documented. When clinical notes explicitly indicated uncertainty (e.g., “unclear history” or “unknown”), the factor was coded as missing to avoid misclassifying.

### Ethical considerations

This database was created for observational purposes, ensuring minimal risks to participants. Informed consent for participation and data sharing was obtained directly from all participants. In cases where individuals were unable to reasonably provide consent themselves, consent was obtained from a parent, guardian, or other legally authorized representative. The Ethics Committee for Research Quality and Ethical Integrity at Clínica Universidad de La Sabana reviewed and approved the consent process as part of the study’s ethical approval (approval number: 021, February 4th, 2020).

## Data Records

This dataset is available on Physionet^19^. The NACeF dataset contains data associated with 768 patients diagnosed with CAP who were hospitalized between January 2020 and July 2022. Data are provided in CVS format, generated through the REDCap platform, and are accompanied by a data dictionary and REDCap XML file to facilitate interpretation and reuse.

**Fig. 2:**
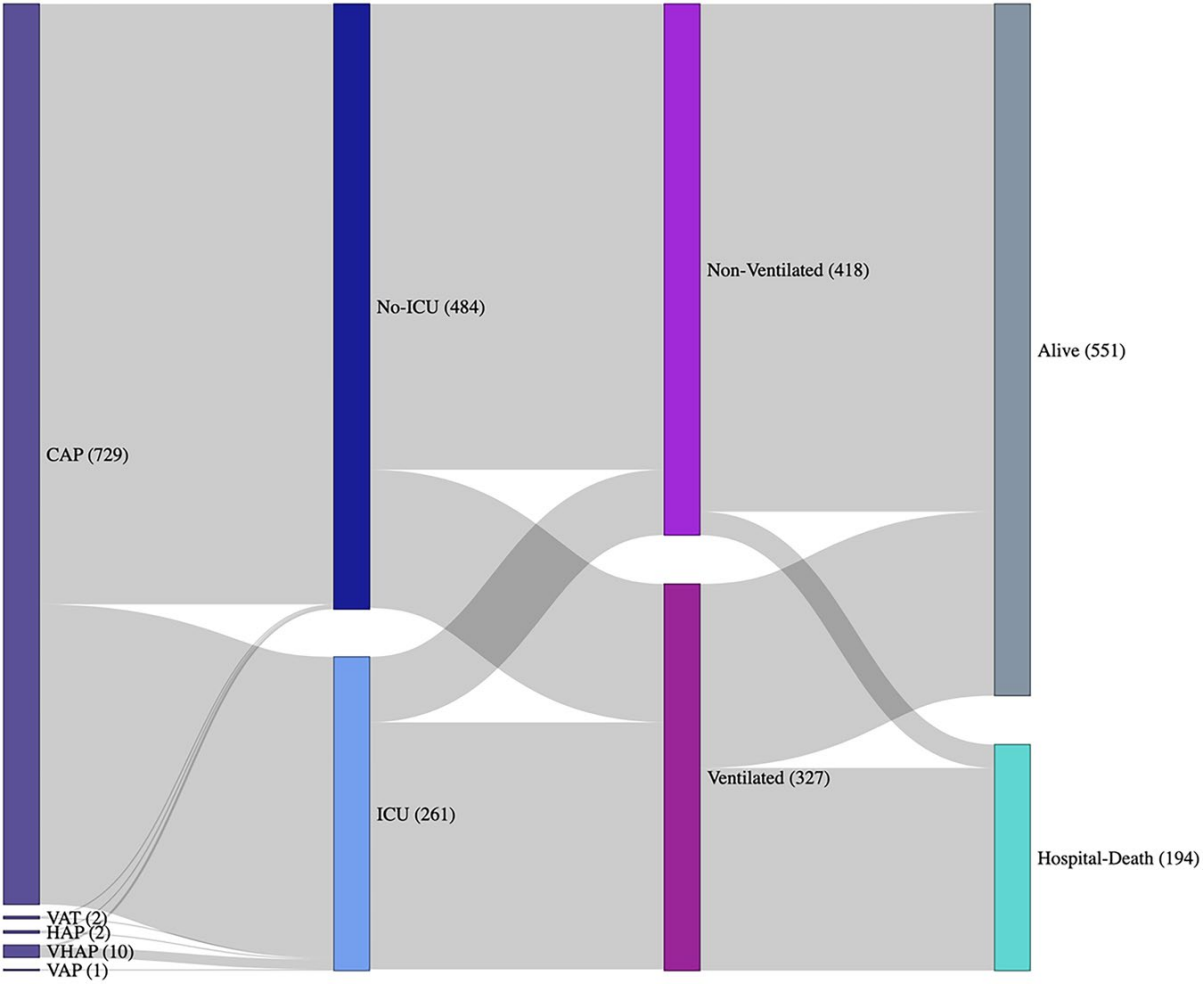
Patients’ characteristics: Main diagnosis, ICU admission, hospital discharge status, and type of ventilation. This figure presents a Sankey diagram illustrating the flow of patients through various clinical stages, including primary diagnosis, ICU admission, hospital discharge status, and type of ventilation. The diagram visually represents the transitions between these stages, with the width of the flows proportional to the number of patients. Starting from the initial main diagnosis categories, the figure shows the subsequent paths leading to ICU admission or non-ICU care, followed by the patient’s discharge status, and finally, the types of ventilation used. This comprehensive visualization aids in understanding the patient journey and the interrelationships between different clinical factors.

The dataset includes patient demographic (age, sex), comorbidities, primary and secondary diagnoses, ICU admission status, severity scores (SOFA, CURB-65, PSI), and details of respiratory support. Of the patients with a recorded primary diagnosis, 97.8% (729/745) had a definitive main diagnosis of CAP. However, a small proportion of patients were ultimately diagnosed with ventilated hospital-acquired pneumonia (VHAP) (1.3%, 10/745), ventilator-associated tracheobronchitis (VAT) (0.3%, 2/745), or healthcare-associated pneumonia (HAP) (0.3%, 2/745) (Fig. 2). These diagnoses were established based on clinical decision and reflect the final documentation in the medical records, rather than superinfections or reclassifications occurring after admission.

**Fig. 3:**
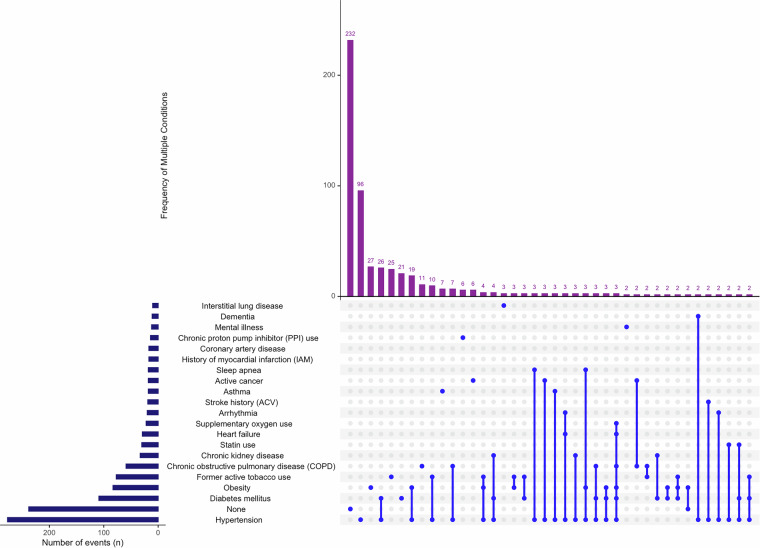
Upset plot, comorbidity patterns in NACef cohort. Using an Upset plot, this figure illustrates the comorbidity patterns observed within the NACef cohort. The plot visually represents the intersection of various comorbid conditions, highlighting the most common combinations of comorbidities in the patient population. This visualization helps identify prevalent comorbidity patterns, which are critical for understanding the complexity of patient health profiles.

**Fig. 4:**
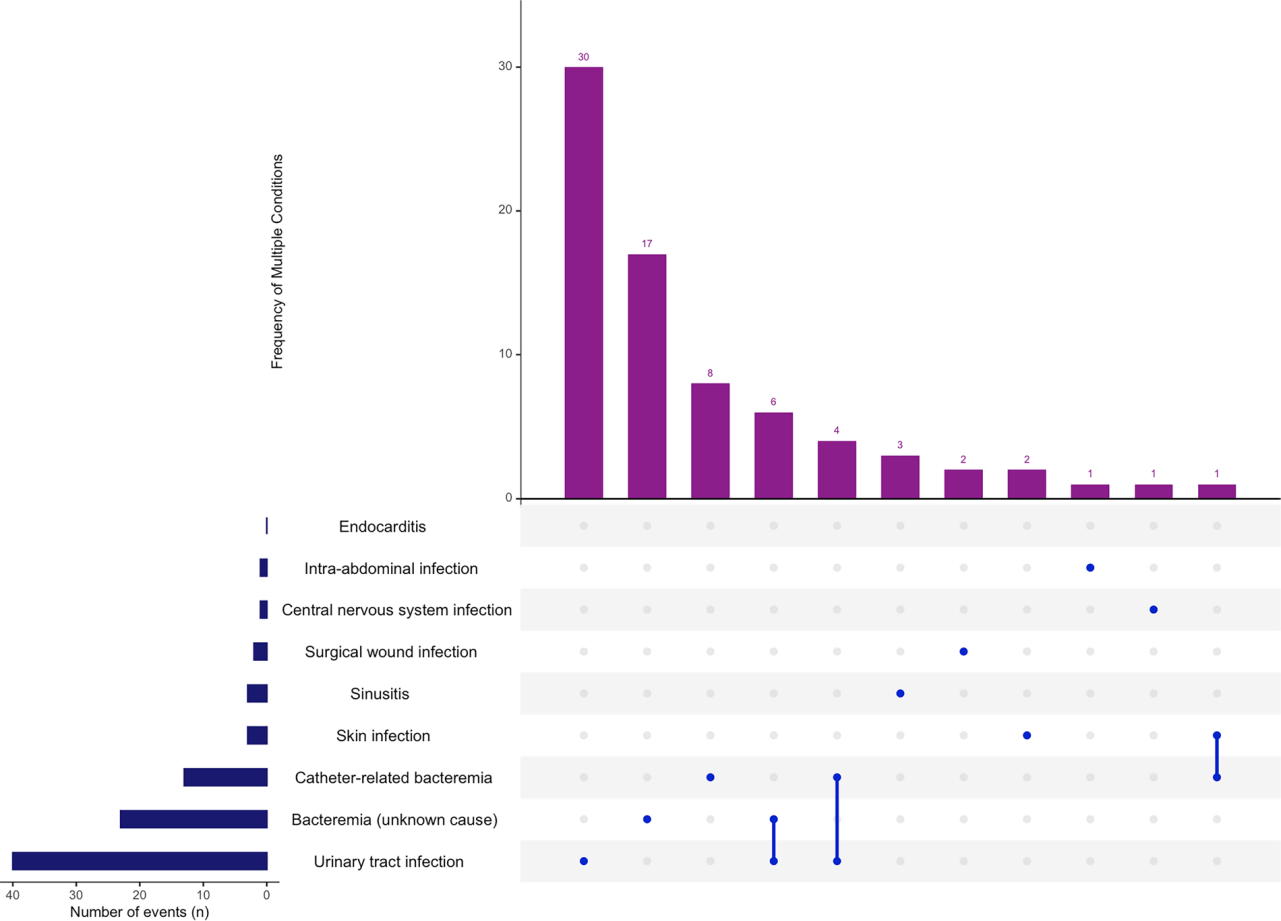
Upset plot, secondary infection patterns in NACef cohort. This figure displays the patterns of secondary infections in the NACef cohort using an Upset plot. It shows the intersections of different types of secondary infections, emphasizing the frequency and combinations of these infections among the patients. This information is essential for recognizing the burden of secondary infections and their impact on patient outcomes.

Comorbidities are included as binary variables and their combinations are illustrated in Fig. 3. Secondary infections confirmed during hospitalization, based on clinical, microbiological, and laboratory findings, are presented in Fig. 4.

Among the patients in the dataset, the mean age is 59 years, with a standard deviation of 14.8 years, and 62.8% (482/768) of the participants are male (Table 1). Overall, 33.9% (261/768) of the patients were admitted to the ICU. At admission, the mean (IQR) values for the SOFA, CURB-65, and PSI scales were 2 (2–4), 1 (0–2), and 76 (57–100), respectively. Within this cohort, 42.6% (327/768) of patients received some form of ventilation support, 81.9% (268/327) received invasive mechanical ventilation, 13.1% (43/327) non-invasive ventilation, and 5.5% (18/327) received high-flow nasal cannula.

## Technical Validation

Multiple data quality checks were performed to determine the accuracy and reliability of the dataset. During data collection, standardized procedures were implemented to guide data entry and minimize error, including structured training for data entry personnel, periodic supervision, and ongoing quality control checks to maintain data integrity. To validate the accuracy of the dataset, experienced clinical researchers conducted a manual review of the electronic medical records, and the REDCap registry was performed by experienced clinical researchers. This process was designed ensure consistency across data sources and to confirm that the collected data accurately reflected clinical reality to the greatest extent possible.

Internal consistency was verified through cross-checking related variables, thus ensuring logical consistency. As part of the data quality and privacy assurance process, we assessed the dataset for extreme values or outliers that could compromise either data integrity or patient confidentiality. To minimize the risk of re-identification, age values greater than 90 years were recoded into a single category (“ < 90”) directly within the REDCap platform during data collection. This modification was part of the predefined de-identification protocol and was applied before data export. These were essential steps to enhance the reliability and usability of the dataset for future and more targeted research and analysis.

## Usage Notes

The online resources provide detailed information about the database and its contents, as well as the custom codes and procedures employed for processing, managing, and organizing the data^19^. Contributions from the research community to share and improve these codes are encouraged. It has been seeded with code that generates patient-level datasets suitable for statistical and machine-learning research, including patient demographics, comorbid conditions at the time of admission, treatments applied, and severity scores. Researchers can directly submit updates, improvements, and additions to the repository via GitHub (https://github.com/TS-ID-CCM/NACef). The repository also includes information on the versions of software used and specific variables or parameters employed to generate, test, or process the current dataset, ensuring reproducibility and transparency in the research process.

This dataset has some limitations. Initially, although we applied standardized clinical criteria to identify patients with CAP, diagnoses were derived from routine clinical documentation without adjudication by an external committee, which may have introduce some degree of misclassification bias.

Second, because the study was based on an analysis of a large clinical database, formal screening information were not available, and we were unable to report the exact number of patients assessed for eligibility, limiting the transparency of the cohort description. However, every single patient admitted to the ICU during the study period were systematically screened to be included in the database. For the same reason, some basic clinical characteristics, such as weight, height and body mass index, were often recorded in unstructured formats or inconsistently documented in the clinical records. To address this limitation, we manually reviewed a subset of clinical records to verify the information, identify systematic issues in documentation, and make corrections if necessary. While we recognize that the completeness of such variables is not ideal, this reflects the reality of working with real-world clinical data. Although we strive for accuracy and completeness, the dataset ultimately mirrors the complexities and imperfections of routine clinical practice.

Third, although no analysis based on this dataset has been published to date, several are currently underway as part of the ongoing research projects. We believe that sharing this national dataset with the scientific community will help identify additional limitations, facilitate validation and encourage broader use.

## Data Availability

The NACef dataset was built using REDCap software and is provided in CVS format to ensure compatibility with common data analysis tools. The anonymized data is available on Physionet^19^ (10.13026/4y3t-pq44), under the PhysioNet Restricted Health Data License 1.5.0. Researchers can obtain access by creating a PhysioNet account and electronically agreeing to the terms of the PhysioNet Credentialed Health Data Use Agreement. Additionally, the REDCap XML, data dictionary, and the codes used to generate descriptive tables in this article are openly available in a public GitHub repository (https://github.com/TS-ID-CCM/NACef).

## Code availability

All code used for data extraction, processing, and cohort creation is openly available in the NACeF GitHub repository: https://github.com/TS-ID-CCM/NACef. The repository is actively maintained, and contributions from the research community are welcomed.
